# Specialized or flexible feed-forward loop motifs: a question of topology

**DOI:** 10.1186/1752-0509-3-84

**Published:** 2009-08-31

**Authors:** Javier Macía, Stefanie Widder, Ricard Solé

**Affiliations:** 1 Complex Systems Lab (ICREA-UPF), Barcelona Biomedical Research Park (PRBB-GRIB), Dr. Aiguader 88, 08003 Barcelona, Spain; 2 Santa Fe Institute, 1399 Hyde Park Road, Santa Fe NM 87501, USA

## Abstract

**Background:**

Network motifs are recurrent interaction patterns, which are significantly more often encountered in biological interaction graphs than expected from random nets. Their existence raises questions concerning their emergence and functional capacities. In this context, it has been shown that feed forward loops (FFL) composed of three genes are capable of processing external signals by responding in a very specific, robust manner, either accelerating or delaying responses. Early studies suggested a one-to-one mapping between topology and dynamics but such view has been repeatedly questioned. The FFL's function has been attributed to this specific response. A general response analysis is difficult, because one is dealing with the dynamical trajectory of a system towards a new regime in response to external signals.

**Results:**

We have developed an analytical method that allows us to systematically explore the patterns and probabilities of the emergence for a specific dynamical response. The method is based on a rather simple, but powerful geometrical analysis of the system's nullclines complemented by an appropriate formalization of the response probability.

**Conclusion:**

Our analysis allows to determine unambiguously the relationship between motif topology and the set of potentially implementable functions. The distribution probability distributions are linked to the degree of specialization or flexibility of the given network topology. The implications for the emergence of different motif topologies in complex networks are outlined.

## Background

Molecular networks in cells are highly complex and dynamic. The global behaviour of these webs and their behavioral patterns are far too complicated to intuitively understand their logic. One way to address this problem is to represent them in terms of simplified interaction graphs combining both biological data and mathematical methods [1-6].

Much effort has been devoted to extract some general features of such networks, dissect them into hierarchical levels, modules and motifs to understand their functionalities, dynamics and evolution [7-16]. Simple switches and oscillators have been shown to map to the core processes of biological decision-making, implemented by two- or three-gene network motifs and characterized by their behaviour around the systems' fixed points [17-22]. However, it is reasonable to think that not only the system's steady state is of interest, but also the way such equilibrium is achieved. Such transient behavior might be characteristic, somehow representing the function performed by the genetic circuitry. In some circumstances, such as in stress responses, a quick change might be favorable [23], whereas in other occasions, e.g. cell-cell intercommunication, it might be more adequate to filter noisy signals and respond only under absolute certainty [24].

**Figure 1:**
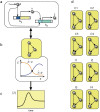
**Schematic representation of the FFL motif and its functions**. Schematic representation of the FFL motif and its functions. In (a) the genetic organization is shown. Activation signal *X *regulates the expression of gene G_*Y*_, whereas gene *G*_*Z *_is regulated in joined mode by gene product *Y *and *X*. This small system can be formally represented in a graph (b) with a given topology and a set of dynamical equations describing the kinetics of its regulatory interactions. For a given set of parameters, a specific type of response (c) might be observed. In (d) all possible regulatory combinations are shown.

Transcriptional networks regulating cell responses exhibit several biochemical wiring patterns, termed network motifs, which appear at frequencies much higher than expected by chance, suggesting that they may have specific functions in the information processing performed by the network. Over the last years, powerful bioinformatic tools such as FANMOD [25] have been developed to detect motif distributions in complex transcriptional networks. One of these motifs is the feed-forward loop (FFL), defined by a transcription factor *X *that regulates a second transcription factor *Y*, such that both *X *and *Y *jointly regulate a target gene *Z *(figure 1a-b). Many examples of FFLs can be found in complex transcriptional networks. For example, in *E. Coli*, FFL is present in the L-arabinose system, where protein Crp is the general transcription factor (*X*) and AraC is the specific transcription factor (*Y*). This motif regulates 40 effector operons in 22 different systems in the network database [26]. A second example can be found in *Saccharomyces *network, where the protein *Mcm1 *(*X*) regulates the expression of *Swi4 *(*Y*). Both proteins *Mcm1 *and *Swi4 *regulate the final expression of *Clb2*. In the yeast network, 39 regulators have been found that are involved in 49 feedforward loops potentially controlling 240 genes [27]. In general, FFLs are known to be associated to multiple key regulations, exhibiting different functionalities, e.g. under conditions of glucose starvation (*CRP*), nitrogen limitation (*rpoN*), and noxious drugs (rob), these regulators act as *X *in a C1 type FFL. On the other hand, I1 type FFLs in yeast include anaerobic metabolism (*HAP1 *as *X*) and nitrogen starvation (*DAL80 *or *GLN3 *as X) systems [28]. In this context, the question about the relation between the functional response implemented by FFLs and their topology arises. The study of the response of three-gene feed-forward loops upon external input shows that they are capable of either implementing transient pulsing (rapid) or, filtering (delayed grader) dynamics [28-35]. However, despite it seems clear that motif topology has an impact on its functionality, is the mapping between motif topology and the possible dynamics one-to-one? Some studies have demonstrated that topology does not necessarily determine function [13,36,37]. Most analysis focused on motif's function have been carried out considering single motif networks. However, recent studies [38,39] have provided evidence that for complex networks, the embedding of the motif with the rest of the network needs to be taken into account.

Here we have developed a method to systematically study the different functions which can be implemented by each FFL motif and how the topology determines univocally the distribution of probabilities for these functions. A will be shown below, this distribution is correlated with the degree of specialization or flexibility of each motif, by taking into account the different likelihood to perform any function. In other words: topology determines the motif's level of functional specialization. Recently, a similar question on the context of genetic clocks has been addressed [40]. The conclusions of the study suggest that for these clocks topology does not determine dynamics univocally. Although our analysis focuses on single motifs, our results provide new insights to understand the different distribution of motifs in more complex networks, as we will discuss later.

In order to analyze the relation between FFL's functionalities and topology we will describe our biological systems in terms of a set of differential equations:

(1)

describing how concentration of different species *Y *and *Z *change during time. Here *Ẏ *and *Ż *represent the derivatives *dY/dt *and *dZ/dt*, respectively. The FFL topology is implicitly described by functions *g*(*X*) and *h*(*X*, *Y*). Assuming that expression of *X *is unregulated, the dynamics of the system can be represented in a two-dimensional diagram displaying *Y *against *Z*, the so called phase space. In the absence of input, the system evolves towards a stable state, i.e. a specific set of values for the concentrations of *Y *and *Z *that remain constant over time. This stable state is determined by the crossing of the so-called nullclines of the system [41] described by the curves *Ẏ *= 0 and *Ż *= 0, i.e. *g*(*X*) = 0 and *h*(*X*, *Y*) = 0 respectively. These curves define the points of the phase space where *Y *and *Z *do not change. The nullclines capture the essence of the dynamical potential of each component and the relevant chemical, physical or biological constraints. Their shapes reflect saturation effects, forbidden ranges of variables or how fast each component responds to perturbations. In other words, a careful analysis of nullclines allows us to understand the dynamics of the underlying systems and its biological implications. The crossings between both nullclines define stable fixed points, where concentrations of both *Y *and *Z *remain constant. Upon input the shape of the nullclines change, providing a new, different stable state. Hence, the system will evolve toward this new state following a trajectory, i.e. a set of intermediate states (*Y*, *Z*) within the available phase space.

As will be shown below, we can analyse the geometrical requirements necessary in order to observe different responses to be generated and the probability for a certain FFL to implement a given function independent of the set of parameters. The main message of our work is that topological interactions encode the shape of the nullclines, which in turn determines the limits of possibilities. The functional response of the FFL depends on the parameter configuration within these limitations. In other words, motif structure does not determine its function, but encodes the probability of potential functions that can be implemented. This paper is organized as follows: We first introduce the general model of the FFL based on ODEs and their respective nullclines. Departing from the nullcline scenarios we determine the constraints imposed onto the dynamics of the response of the FFL. Finally, these constraints are formalized analytically in such a way that all feasible types of behaviour can be evaluated. This evaluation allows for the first time to draw generally valid conclusions on the relation between motif topology and function in FFLs.

## Results and Discussion

### General model of the FFL

The analysis is focused on the most general FFL formed by three genes. We assume that the gene circuit acts as a functional unit responding to an external input by producing output. In figure (1) we show an schematic representation of the FFL, depicting three genes *G*_*X*_, *G*_*Y *_and *G*_*Z*_, with regulatory interactions among each other via their corresponding proteins *X*, *Y *and *Z*.

Gene *G*_*X *_(not shown) has a constant production of its protein which is independent of the regulation of the other proteins. We assume that *X*, however, is synthesized in its inactive form and needs an external effector (the input of the circuit) for its activation. The concentration of active *X *without input is negligible. Upon addition of effector, the activation of *X *proceeds very rapidly compared to synthesis and decay and hence, can be approximated by a step function

(2)

as in [28]. In the following, *X *will denote the active protein. Transcription of *G*_*Y *_and *G*_*Z *_is subjected to internal regulation. Production of *Y *depends on regulator *X*, whereas production of *Z *depends on both *X *and *Y*. The FFL can be described by the following ODEs

(3)

(4)

The parameter *γ*_*i *_represents the basal production of protein *i*, where *i *= {*X*, *Y*, *Z*}. In this parameter we subsume the concentration of all biochemical elements which remain constant in time. The degradation rate of protein *i *is denoted as *d*_*i*_. The binding equilibrium of the regulators *j *with the gene *G*_*i *_are denoted by , with *j *= {*X*, *Y*, *Z*}. The tunable positive parameters *α*^*X *^and *β*^*j *^describe the type of regulatory interactions, i.e. activation or inhibition, for gene *G*_*Y *_and *G*_*Z*_, respectively, without any predefined specific assumptions. They provide the regulatory rates with respect to the basal transcription. Values < 1 correspond to inhibitory regulation, whereas > 1 accounts for activation. The parameter *β*^*XY *^accounts for the simultaneous regulation of *G*_*Z*_.

Traditionally, studies on FFL dynamics have been performed under the assumption of Boolean logic [28,42] for the control of the output regulation. The presented model includes all theses cases as specific subsets of parameters. For example, assuming a Boolean AND logic for a circuit, where the output *Z *is positively regulated by *Y *and *X*, is described by the following parameter configuration: *β*^*x *^= *β*^*Y *^= 1, *β*^*XY *^> 1 and . The same circuit displaying OR logic requires *β*^*x *^= *β*^*Y *^> 1, *β*^*XY *^= 0 and  = 0.

Finally, *n *and *m *denote the degree of multimerization of the regulators. The presented results, however, are considering the general case independent of the size of the regulatory factors.

### Nullclines' analysis: Changes of the nullclines upon input

The system's fixed points can be determined by inspection of the crossings of its nullclines. The studied input-output system will remain stable unless an external input pushes the FFL towards another stable equilibrium. The FFLs dynamical response upon this change depends on a specific configuration of circuit parameters. Notwithstanding, the possible response-dynamics are restricted by the shape of the nullclines. The general expressions for the nullclines are obtained imposing *Ẏ *= 0 and *Ż *[41] and written as follows:

(5)

(6)

For a general analysis of the nullclines' shape independent of the specific parameters and the size of the multimeric regulators, we focus on a simple set of geometrical features. Within the phase space spanned by {*Y*, *Z*}, the nullcline (5) is a simple, vertical straight line. Nullcline (6) shows an horizontal asymptote located at

(7)

and crosses the vertical axis at

(8)

**Figure 2:**
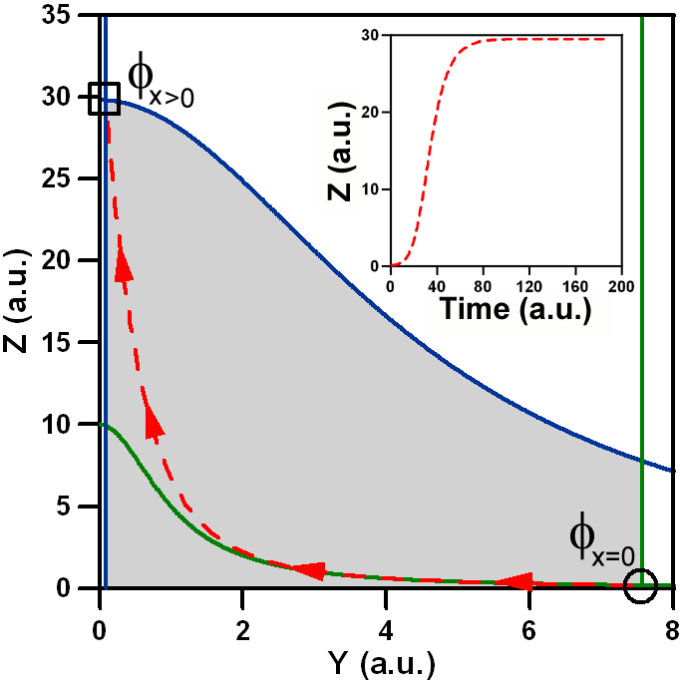
**Input effects on the nullclines**. The change of the nullclines upon input is illustrated in these examples. The green lines are the nullclines (5) and (6) without input crossing at point *ϕ*_*X *= 0_. In absence of input, the system is stable in this point. Upon input the nullclines change and are depicted as blue lines, shifting the stable point to *ϕ*_*X *> 0_. The change of the fixed point, forces the system to evolve towards the new regime. The red dashed line represents its trajectory in the phase space. The inset shows the corresponding time course of the system's output. The parameters of the simulations are: *γ*_*Y *_= 1, *α*^*X *^= 0,  = 100, *d*_*Y *_= 0.1, *γ*_*Z *_= 1, *β*^*X *^= 3,  = 100, *β*^*Y *^= 0,  = 1, *β*^*XY *^= 0,  = 100 and *d*_*Z *_= 0.1. In absence of input *X *= 0 and in presence of input we consider *X *= 1.

**Figure 3:**
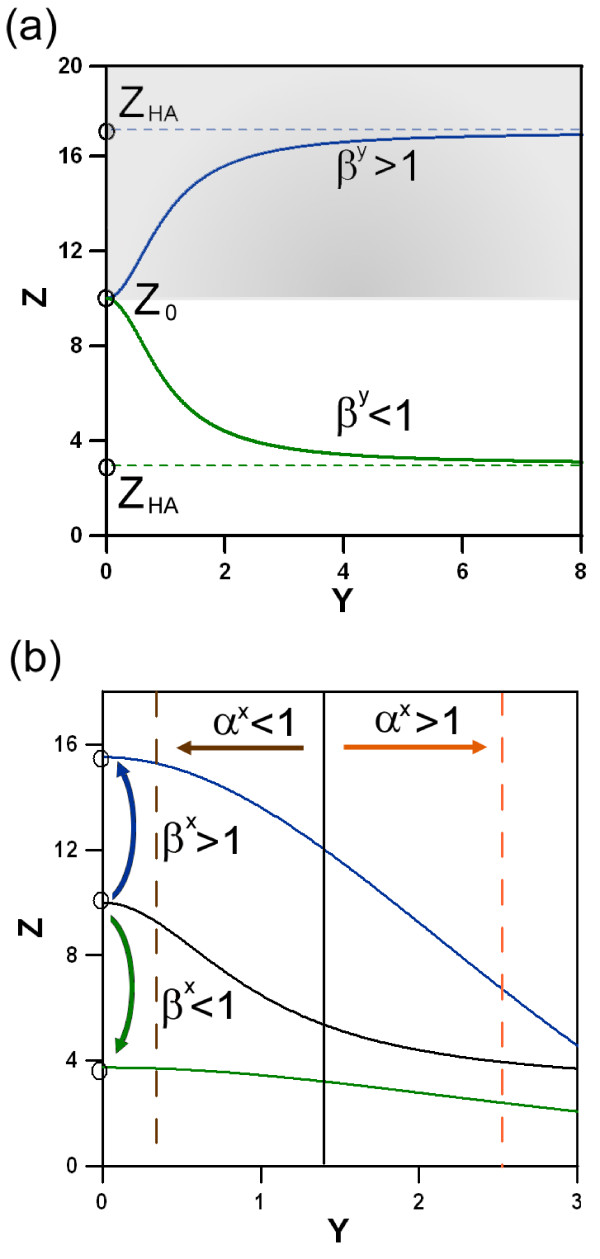
**The effects of the regulatory parameters on the geometry of the nullclines**. In (a) we show the impact of positive (grey area) or negative (white area) regulation of *G*_*Z *_by *Y*. The geometrical effect associated to *β*^*Y *^is the only independent of presence of input. In (b) we depict input-dependent effects associated to other regulatory parameters. Nullcline (5) shifts are related to the regulatory interaction of *X *upon *G*_*Y*_. The orange line represents the qualitative location for *α*^*X *^> 1, in brown the location for *α*^*X *^< 1 is shown. Similarly, the crossing point of nullcline (6) with the vertical axis is shifted to higher (blue) or lower values (green) depending on the type of regulation by *β*^*X*^.

Furthermore, expression (6) shows a single inflection point, i.e. a point where curvature changes from concave to convex or viceversa, in the biological domain defined by *Y *≥ 0 and *Z *≥ 0, but no extrema (local maximum or minimum). In order to understand how the input triggers the dynamical response, we study the configuration of the two nullclines with *X *= 0, i.e. no input, versus *X *> 0, i.e. with input. In figure (2) we show a numerical example. In absence of input, the system is governed by a single stable fixed point, denoted by *ϕ*_*X *= 0_, located at the crossing of the nullclines (5) and (6). On addition of input, the fixed point moves, because the nullcline configuration changes. Now the system shifts from *ϕ*_*x *= 0 _to the new stable fixed point *ϕ*_*x *> 0_. The point *ϕ*_*X *> 0 _is determined by the crossing of the new location of the nullclines for *X *> 0. The dynamical response of the system, which is represented by the trajectory of the FFL in phase space, is generated by this change of the stable regime. A subset of four parameters, i.e. {*α*^*X*^, *β*^*X*^, *β*^*Y*^, *β*^*XY*^}, describing the interactions of the proteins, classify the type of the circuits into coherent and incoherent according to [28]. Without input, only *β*^*Y *^is relevant for the geometry of the nullcline *Ż *= 0. If *β*^*Y *^> 1 the nullcline rises, because *Z*_*HA *_> *Z*_0_, whereas for *β*^*Y *^< 1 the nullcline decreases as shown in figure (3a). Under the presence of an external input the other parameters become relevant (figure (3b)):

1. The regulation of *Y *by *X*, described by *α*^*X*^, defines the direction of the shift of the straight nullcline (5) regarding its earlier position for *X *= 0.

2. The regulation of *Z *by *X*, described by *β*^*X*^, displaces the crossing of the nullcline (6) with the vertical axis to a higher (*β*^*X *^> 1) or a lower (*β*^*X *^< 1) value.

3. Finally, the joint regulation *β*^*XY *^of *Z *by *X *and *Y*, strongly influences the location of the horizontal asymptote *Z*_*HA*_.

As we will see in the following sections the geometrical features generated by the biological interactions will form the basis for the dynamical behaviour of the FFL.

### Response of the FFL

**Figure 4:**
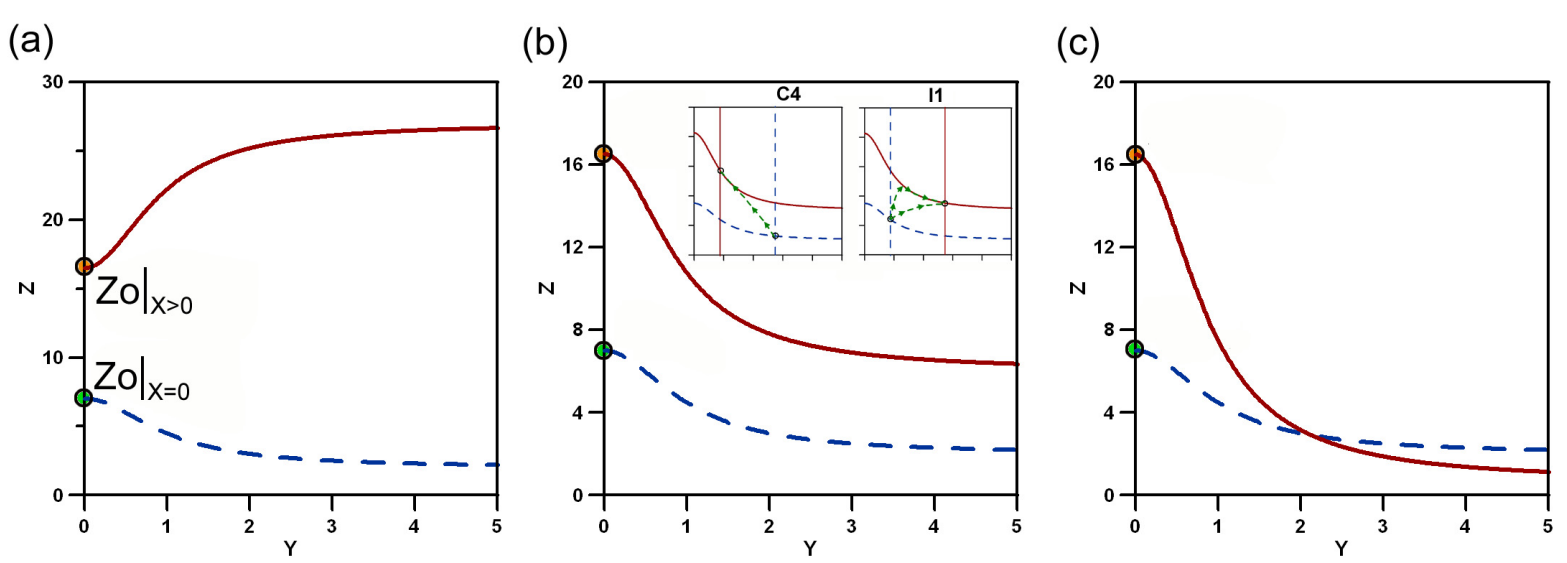
**Possible shapes of nullcline**. The set of possible shapes for the nullcline *Ż *= 0 with (brown) and without (blue) input. Figures (a), (b) y (c) correspond to different biological regulations. In (a) the joined regulation by proteins *X *and *Y *enhances the production of the output *Z *(*β*^*XY *^> 1). In (b) and (c) the production of *Z *is reduced (*β*^*XY *^< 1), but in (b) the reduction is smaller due to *β*^*XY *^> *β*^*Y*^, whereas in (c) we apply strong inhibition *β*^*XY *^<*β*^*Y*^. Insets in (b) show the feasible trajectories for C4 and I1, respectively. For C4 the functionality of the trajectory is uniquely defined as grader, whereas I1 can implement pulser or grader dynamics, depending on the specific parameters.

To understand how the changes in the nullclines upon input confine the feasible dynamical response of the circuit, we will focus our attention on two representative cases, namely a coherent and an incoherent type ([28]). We choose circuits C4 and I1, respectively, which differ only in one regulatory interaction in *G*_*Y*_, i.e. *α*^*X*^. Due to this interaction only nullcline (5) is affected and with it the direction of its shift as can be seen in figure (3b). The other interactions are the same for both circuit architectures (*β*^*x *^> 1, *β*^*Y *^< 1) and hence the possible nullcline's shapes associated to expression (6) are also the same. In figure (4) we show their subset of changes realized by the nullcline. Based on the positive interaction of *β*^*X *^the crossing point *Z*_0_|_*X *> 0 _with the vertical axis always shifts toward higher values for cases with input compared to cases without input (*Z*_0_|_*X *= 0_).

Two biological scenarios for the joint regulation of *G*_*Z *_by *Y *and *X *are plausible: either the complex acts as an activator *β*^*XY *^> 1 as shown in figure (4a) or as inhibitor *β*^*XY *^< 1. In (4b) we show the scenario associated with the conditions *β*^*XY *^< 1 and *β*^*XY *^> *β*^*Y*^. The nullcline moves down, but does not cross the original nullcline. In (c) conditions *β*^*XY *^< 1 and *β*^*XY *^<*β*^*Y *^lead to a single crossing. The same conditions can lead to a double crossing of the nullclines, however our numerical analysis (data not shown) indicates that the probability to find an adequate configuration of parameters has very low probability (< 0.3%). For simplicity we discard these cases (see Methods for a detailed example of our analysis applied to nullclines with double crossing). By using the relatively simple configuration of the nullclines shown in (b) we already find different possible behaviour of the two circuits C4 and I1 due to their opposing *α*^*X*^.

Whereas for C4 the nullcline's (5) shifts to higher values, allowing only for grader trajectories (inset 1), I1 may show instead two different functionalities. The shift direction is to lower values and hence either grader or pulser can be implemented depending on the set of parameters (see inset 2). The fact that a range of feasible functional scenarios can be intuitively deduced, demands for a method of unambiguous discrimination to resolve the problem.

#### Separatrix

**Figure 5:**
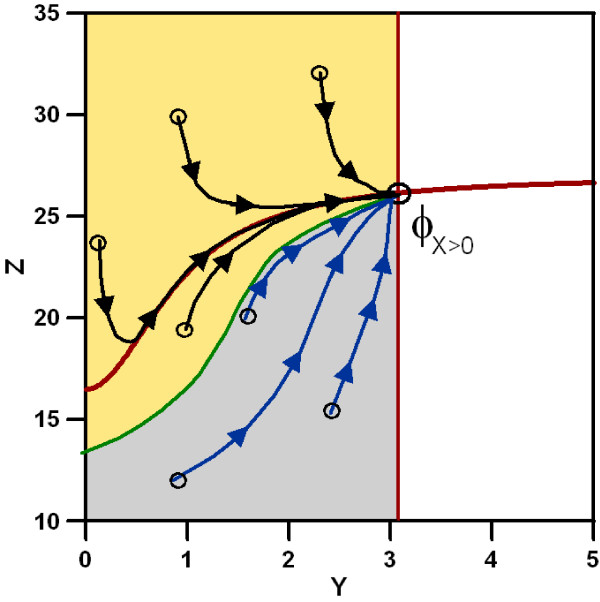
**Schematic representation of two areas characterizing the behaviour of the trajectory**. The nullclines of the system with input are depicted in brown. Any trajectory starting in the pink area will reach the nullcline *Ż *= 0 first and then follow its path to the fixed point *ϕ*_*X *> 0_. Any trajectory starting in the blue area will reach the fixed point directly without intersecting the nullcline first. The frontier between these two regions, the separatrix, is drawn in green. As example some trajectories have been depicted to illustrate their qualitatively different course.

**Figure 6:**
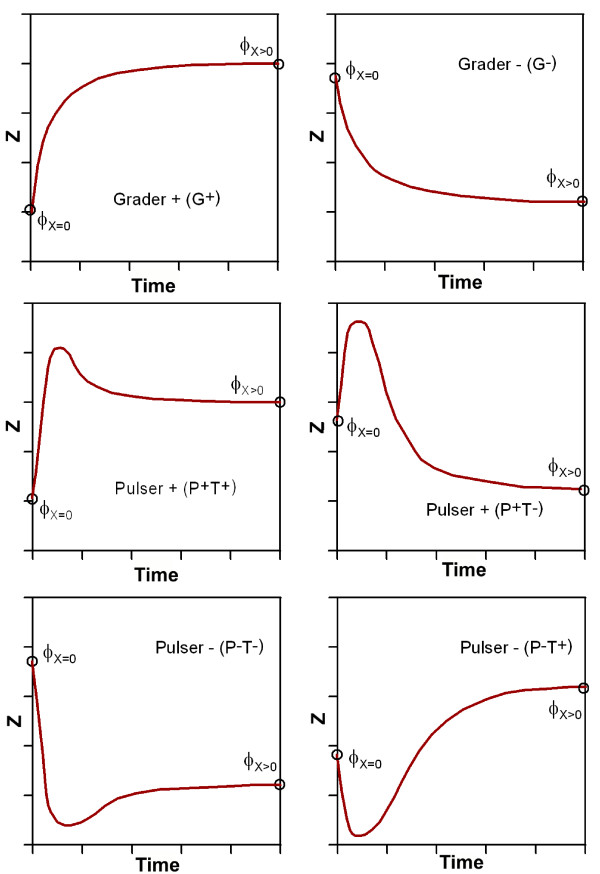
**Qualitative representation of all possible timecourse responses realized by FFLs**. Here, 'G' indicates grader and 'P' pulser response of the circuit. The initial slope is represented by '+' or '-' in our nomenclature. The difference of the initial versus the target concentration is denoted as '*T *+' or '*T*-'.

Sometimes, two qualitatively different dynamics are possible for a given set of nullclines (figure (4b)). In that case, if we consider any arbitrary initial point (*Y*, *Z*) in phase space (i.e. any arbitrary concentration values of *Y *and *Z*) phase space can be divided into two different areas. As shown in figure (5) this partition of phase space is characterized by a different functional behaviour of the trajectories upon input: starting in one part (yellow area) the trajectory will first join/intersect the nullcline *Ż *= 0 and then following the nullcline until it reaches the fixed point. Starting from an arbitrary point in the other region (grey area) the trajectory will only reach the nullcline in the fixed point *ϕ*_*X *> 0_. This qualitative difference in the trajectories gives rise to a different functional behaviours of the circuit (see figure (6)).

**Figure 7:**
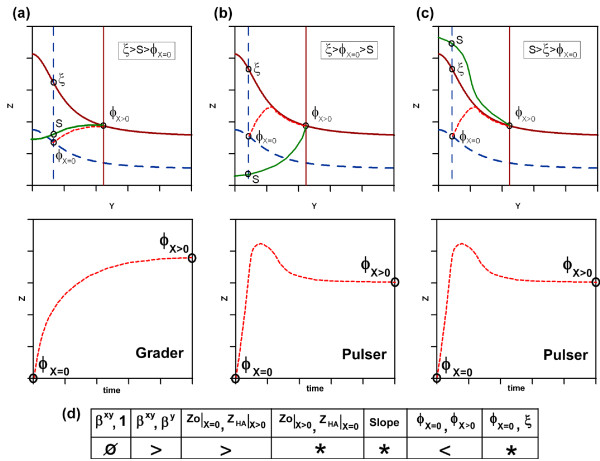
**Positional effect of the separatrix**. Here we illustrate the positional effect of the separatrix *S*, *ξ *and *ϕ*_*X *= 0 _on the functional behaviour of the FFL (a-c). The relative position between the three crossing points with the vertical nullcline *Ẏ *= 0 without input determines the functionality of the time course. Positioning shown in (a) results in grader, whereas (b) and (c) display pulser dynamics. The respective relative location is outlined in the boxes. In (d) the unique backbone of parametric requirements defining univocaly the shape of the nullclines is shown. Each column describes the relation (> or <) between two characteristic elements (head column). The single values are described in the text. Column 'Slope' refers to the slope of the nullcline *Ż *= 0 with input (*X *> 0) and can be positive or negative. The columns containing '∅' or '*' do not need to be explicitly addressed, because of parametric inter-dependencies (see text).

The system without input is determined by its fixed point *ϕ*_*X *= 0_. The circuit will exhibit a given dynamic behavior depending on which part of phase space *ϕ*_*X *= 0 _is located. The location of the boundary between the two dynamical areas, termed separatrix, allows classifying the functionality of the trajectory. The key elements for the discrimination of the different possible functionalities, for a given geometry of the nullclines, is the relative position of three points: the crossing of the vertical nullcline *Ẏ *= 0 without input, with: i) the nullcline *Ż *= 0 without input (*ϕ*_*X *= 0_), ii) the separatrix (*S*) and with iii) the nullcline *Ż *= 0 with input (*ξ*). We illustrate this with an example (circuit I1) in figure (7a-c) where the relative positions of the crossing points and their implication for the time course is outlined. An analytical estimation of the separatrix and all the possible combinations of these elements with its resulting dynamics are presented in Supplementary Materials.

Our analysis can be generally applied to all three-gene FFLs. We have shown that both examples show enough plasticity in the dynamics to implement different type of response. In the next section we will focus on the probability of emergence of the different feasible types of dynamics.

### Probability of emergence of different FFL's dynamics

In the previous section we have shown that in both examples, C4 and I1, more than one possible dynamic behavior can be obtained depending on the specific set of parameters chosen. Generally we can list six types of dynamics, namely positive or negative graders (*G*^+^, *G*^-^) and positive or negative pulsers (*P*^+^, *P*^-^). Grader response corresponds to an increase (+) or decrease (-) of the concentration of *Z *characterized by a transient from the initial to the final state where the concentration of *Z *never is higher (*G*^+^) or lower (*G*^-^) than the final concentration. In general, grader responses are related with responses able to filter noise and respond only on absolute certainty [24]. On the other hand, a pulser response is characterized by a rapid increase (*P*^+^) or decrease (*P*^-^) of the concentration of *Z *reaching higher (+) or lower (-) values of *Z *before they reach the final state. Note that for the pulsers independently of the pulse direction (*P*^+ ^or *P*^-^) the final concentration of output protein *Z *can be higher (*T*^+^) or lower (*T*^-^) than the initial concentration. Hence we separately analyze four different pulser dynamics, namely *P*^+^(*T*^+^), *P*^+^(*T*^-^), *P*^-^(*T*^+^), *P*^-^(*T*^-^). The time courses of the different functionalities are outlined in figure (6). A specific subset of dynamics can be determined for each FFL frequently containing functions, which cannot be implemented. Based on this results the main question is: Are all the feasible dynamics equally probable? To address this question we have performed an analytical study of the parametric requirements necessary to implement a given type of dynamical response.

#### Backbone of requirements for the FFL response

We can deduce geometrically all possible sets of trajectories. We discriminate the necessary parametric conditions for the emergence of one specific dynamic among all the possible, due to the relative position of the initial point *ϕ*_*X *= 0 _of the trajectory in relation to the separatrix *S*, *ξ *and the specific shape of the nullclines. The parametric conditions, which determine the shape of the nullcline, can be systematically formalized. The key geometrical elements of the nullclines can be described by a set of exclusive parametric combinations defined in a string, which we call the *backbone of requirements*. Each position in this sequence contains the solution for two possible parametric states.

The example shown in figure (7) is meant to illustrate the procedure. The cases in (a-c) display the same geometrical shape and can be described by a single backbone of requirements shown in (d). Note that not all the elements of the parametric sequence need to be defined explicitly. In certain occasions some of them are uniquely defined by previous conditions, denoted by *, or otherwise do not have an impact on the geometrical scenario, represented by '∅'. For example position one is denoted '∅'. If condition two (*β*^*XY *^> *β*^*Y*^) is satisfied, position one is not relevant, because both solutions (*β*^*XY *^> 1 or *β*^*XY *^< 1), provide the same geometry. On the other hand position four, denoted as '*', is always solved as '>', because it can be deduced from the second condition given that *β*^*X *^> 1.

#### Quantification of the dynamical probabilities

Within the framework of our model, we assume that the feasibility of given dynamical behaviour (transient response) is heavily dependent on the number of exclusive, parametric configurations necessary for the realization of this behaviour. These are subsumed in the backbone of requirements. We have generated all possible backbone sequences for the circuits C4 and I1. For each of these sequences the separatrix discriminates among different dynamics as we have shown in figure (7a-c). In circuit C4 we have found 18 different sequences implementing five different types of dynamics, where as for circuit I1, 21 sequences implement four different behaviors. The detailed list of backbone sequences are shown in Supplementary Materials. The total number *N*_*C *_of possible requirements captured within one sequence *i *within a circuit *k *= {*C*4, *I*1} is constant and follows the sum:

(9)

Here,  represents the number of requirements having no impact on the nullclines' geometry. For this kind of elements of the sequence, both possible solutions ('>' or '<') are valid and hence discrimination is unnecessary. Therefore,  different combinations of parameters are described by the same backbone sequence providing the same geometry for the nullclines. The second term , is the number of elements predefined by other conditions of the backbone. Finally,  is the number of requirement actually necessary to determine univocaly the shape of the nullcline. In other words, in order to implement a determined backbone sequence it is sufficient to properly establish the conditions .

Once these conditions are set, the rest of the sequence is determined. This allows to calculate the probability for a given set of parameters to implement a certain backbone sequence *i *as

(10)

For a given FFL topology it is possible to find different sets of parameters compatible with such topology but implementing different dynamics among the six possibilities. Each set of parameters fits in a specific backbone sequence. For each backbone sequence there exist three different relative positions of the separatrix discriminating among the different possible dynamics. This idea is illustrated in figure (7). The same backbone sequence shown in 7d can implement two different dynamics, namely *G*^+^and *P*^+ ^*T *^+^. The location of the separatix shown in figures (7a-c) depends on the numerical values of the parameters. If these specific values locate the separatrix as depicted in figure (7a) the FFL motif will implement a grader dynamics, whereas other locations (see figures (7b, c)) will provide a pulser dynamics. The probability of circuit *k *to implement a given dynamic *j *= {*G*^+^, *G*^-^, *P*^+^(*T*^+^), *P*^+^(*T*^-^), *P*^-^(*T*^+^), *P*^-^(*T*^-^)} described by the backbone sequence *i *can, thus, be calculated as:

(11)

where Ψ_*ijk *_is the number of equal dynamical outcomes *j *for a given backbone *i *in the *k*_-*th *_FFL and *T*_*jk *_the total number of backbone sequences implementing dynamic *j*. The normalization constant Ω_*k *_is defined by imposing the condition  and gives:

(12)

**Figure 8:**
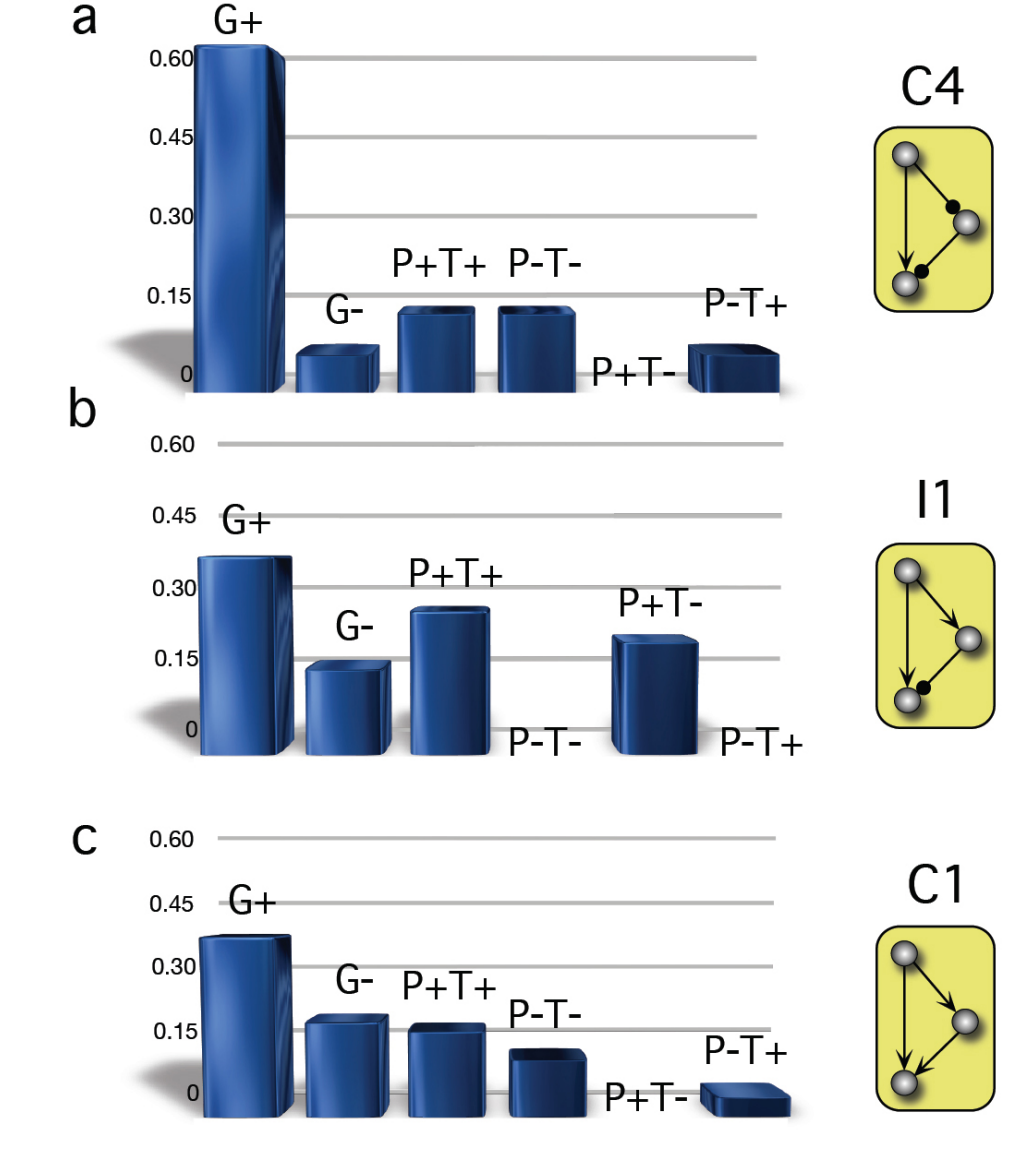
**The probability for emergence of specific functionalities**. In (a) the distribution for the coherent FFL C4 is shown. The probability to implement *G*^+ ^dynamics is significantly higher than to implement any other possible function. In (b) we plot the distribution of the incoherent FFL I1. All possible functions range at similar probability values. The two FFLs differ in a single regulatory interaction.

By calculating the probability of implementation of the different types of dynamics for the different FFLs we obtain an interesting picture regarding our initial question, whether the topology of the FFL implements its function. As shown in figure (8a) the coherent FFL C4 implements five different types of dynamics. Their probability (given an arbitrary set of parameters) is however very different. We find that C4 is significantly specialized for *G*^+ ^dynamics. The degree of specialization is equally reflected in its robustness versus parametric change. On the other hand, the incoherent FFL I1 potentially implements four different types of dynamics, as shown in figure (8b). The probabilities for I1 to implement these functionalities are within the same range, unlike in C4. In figure (8c) the distribution of probabilities of C1 (the most abundant FFL) obtained performing the same analysis is shown. The specific topology allows for flexibility at the cost of less specialization. We see that the topology of the FFL does not implement its function, but instead the probability of a certain function to arise. If, for a given FFL, the distribution accentuates a certain function, the FFL is said to be specialized for this function. If the probabilities are located within the same range, the topology implements flexibility. Both aspects are equally relevant in terms of adaptation and evolvability.

The highest level of specialization of a given motif would correspond to a distribution of probabilities displaying a single peak, whereas the maximum level of flexibility would correspond to a flat distribution of probabilities, where all the potential dynamics would be equally probable. In order to compare the level of specialization of different FFL topologies we propose to measure the *peakedness *of the probability distribution, i.e. the kurtosis [43] (see Methods for details about kurtosis). The values of kurtosis are K_*C*1 _= 4.6, *K*_*C*4 _= 7.8 and *K*_*I*1 _= 1.28, respectively indicating that C1 has an intermediate level of specialization with respect to C4 (more specialized) and I1 (more flexible). The same qualitative distributions are obtained by numerical simulation choosing random sets of parameters and counting the frequency of each dynamics (data not shown).

## Conclusion

FFL motifs appear frequently in cellular regulatory networks. Despite the efforts devoted to understand how FFLs encode their functionalities, the question about the relation exact between function and topology remained open. In this work we have presented a new analytical formalism based on the geometric analysis of the system's nullclines to elucidate this question. We found that the dynamical response triggered by the external input can be analysed in terms of: i) the nullcline's geometry as described by a backbone sequence of parametric conditions and ii) the specific location of the initial stable state in the phase space with respect to the nullclines and the separatrix. This puts us into the position to unambiguously enumerate the probability of a given FFL to implement a certain function. Our results support this view topology does not define a unique functionality, as have been previously discussed, ([36]). Circuits with the same topology can implement different functions, yet not all of these possible dynamics are equally probable. However, topology defines univocally the distribution of probabilities for the emergence of the different feasible responses.

For illustrative purposes we have analyzed two interesting examples, namely circuits C4 and I1, exhibiting the same regulatory interactions except a single regulation of the gene *G*_*Y *_by the protein *X*. In these cases we found two paradigmatic scenarios: circuit C4 can implement *G*^+ ^response with significantly higher probability than the other feasible dynamics, whereas, I1 exhibit more uniform distribution of probabilities. These results demonstrate that C4 has a specialized topology, optimal for the implementation of grader response, whereas I1 has a high degree of flexibility among different dynamics. Under an evolutionary perspective, a trade-off between these different features, flexibility and specialization, is likely to play an important role. This problem will be investigated elsewhere.

In single motif networks a given function will be implemented with high probability by the most specialized topology. However, in evolved, complex networks other aspects need to be considered. In order to obtain reliable networks, i.e. robust and with high fault tolerance, complex topologies can emerge as a result of the evolutionary process. An evolved and fit network is not necessarily the sum of its optimal sub-modules. In order to provide redundancy and degeneracy, flexible sub-modules, able to change their functionalities with minimal cost are often a good solution to reliability [44]. Future work will be devoted to analyze the implications of these two characteristics in the natural emergence of current biological networks.

## Methods

### Analytical estimation of the separatrix

The unique discrimination of the circuit's dynamical behaviour is determined by the location of the initial point *ϕ*_*X *= 0 _with respect to the nullclines and the separatrix. The separatrix defines the boundary between two different areas defining the dynamical outcome in phase space. If the initial point of the trajectory lies within one part (in the following *A*_1_) it will reach the nullcline *Ż *= 0 directly at the fixed point (*ϕ*_*X *> 0_). If it lies in the other part (*A*_2_) the trajectory will reach the nullcline before the fixed point and follow its path towards the final state. In order to obtain an analytical expression for the separatrix of phase space, we examine any arbitrary point and its corresponding vector field described by the system's ODEs (see article). Each arbitrary point is defined by its two components (*Y*, *Z*) in phase space, as is the final point *ϕ*_*X *> 0 _= (*Y*_*f*_, *Z*_*f*_). The relation between the horizontal distance *Y*_*f *_- *Y *and the horizontal component of the field *Ẏ *and the corresponding vertical elements determine the area. We assume that each point (*Y*, *Z*) in one area satisfies the local condition

(13)

whereas the opposite is true for each point in the other area. The separatrix is defined by the set of points satisfying

(14)

Using the system's ODEs we obtain the following expression for the separatrix:

(15)

In order to test this expression we have performed numerical simulations with random sets of parameters and multiple random initial points. This separatrix expression defines properly the frontier between *A*_1 _and *A*_2_.

### Relation between dynamics and geometrical elements

The relation between the geometrical elements *S*, *ξ *and *ϕ*_*X *= 0 _and the slope of the nullcline *Ż *= 0 upon input defines the function of the FFL. The combinations are summarized in tables (1) and (2).

**Table 1:** Relation between geometry of the nullclines and functionality

Sl.	Relation	Function	Relation	Function
+	*S *> *ϕ*_*X *= 0 _> *ξ*	*P*^-^	*ξ *> *S *> *ϕ*_*X *= 0_	*G*^+^

-	*S *> *ϕ*_*X *= 0 _> *ξ*	*G*^-^	*ξ *> S > *ϕ*_*X *= 0_	*G*^+ ^if *Z*_*f*_* > Z*_*i *_else impossible

+	*ϕ*_*X *= 0 _> *ξ *> *S*	*P*^-^	*ξ *> *ϕ*_*X *= 0 _> *S*	*G*^+^

-	*ϕ*_*X *= 0 _> *ξ *> *S*	*G*^-^	*ξ *> *ϕ*_*X *= 0 _> *S*	*P*^+^

+	*ϕ*_*X *= 0 _> *S *> *ξ*	*G*^- ^if *Z*_*f *_<*Z*_*i *_else impossible	*S *> *ξ *> *ϕ*_*X *= 0_	*G*^+^

-	*ϕ*_*X *= 0 _> *S *> *ξ*	*G*^-^	*S *> *ξ *> *ϕ*_*X *= 0_	*P*^+^

Possible combinations of nullcline and geometrical elements for the evaluation of the FFL functionality considering *α*^*Y *^> 1. 'Sl.' denotes the slope of the nullcline *Ż *= 0 upon input, *Z*_*f *_is the concentration of protein Z in its equilibrium *ϕ*_*X *> 0 _and *Z*_*i *_its concentration at the initial point *ϕ*_*X *= 0_.

**Table 2:** Relation between geometry of the nullclines and functionality

Sl.	Relation	Function	Relation	Function
+	*S *> *ϕ *_*X *= 0 _> *ξ*	*G*^-^	*ξ *> *S *> *ϕ*_*X *= 0_	*G*^+ ^if *Z*_*f *_* > Z*_*i *_else impossible

-	*S *> *ϕ*_*X *= 0 _> *ξ*	*P*^-^	*ξ *> *S *> *ϕ*_*X *= 0_	*G*^+^

+	*ϕ*_*X *= 0 _> *ξ *> *S*	*G*^-^	*ξ *> *ϕ*_*X *= 0 _> *S*	*P*^+^

-	*ϕ*_*X *= 0 _> *ξ *> *S*	*P*^-^	*ξ *> *ϕ*_*X *= 0 _> *S*	*G*^+^

+	*ϕ*_*X *= 0 _> *S *> *ξ*	*G*^-^	*S *> *ξ *> *ϕ*_*X *= 0_	*P*^+^

-	*ϕ*_*X *= 0 _> *S *> *ξ*	*G*^- ^if *Z*_*f *_<*Z*_*i *_else impossible	*S *> *ξ *> *ϕ*_*X *= 0_	*G*^+^

Possible combinations of nullcline and geometrical elements for the evaluation of the FFL functionality considering *α*^*Y *^< 1.

### Analysis of the relaxation dynamics after induction

**Figure 9:**
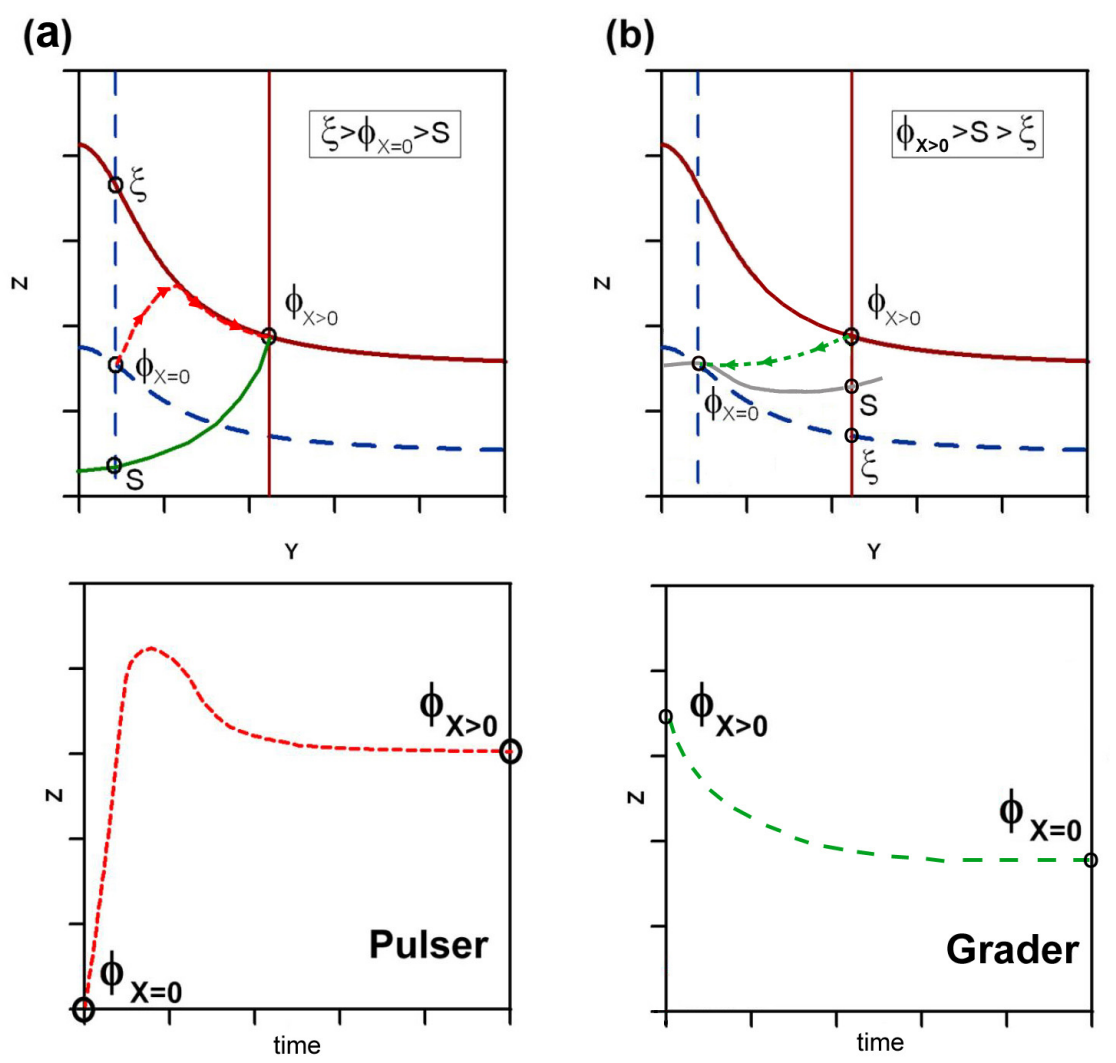
**Schematic example of induction and relaxationdynamics**. In (a) the system evolves from the initial stable state *ϕ*_*X *= 0 _to the final state *ϕ*_*X *> 0 _upon addition of an external input. In this case the geometrical constraints of the nullclines upon input (brown) allow for a pulser dynamics (*P*^+^*T*^+^). In (b) after input deletion the system evolves from *ϕ*_*X *> 0 _to *ϕ*_*X *= 0_. The relaxation dynamics is determined by the shape of the nullclines without input (blue). Here the dynamic is grader (*G*^-^). The same mathematical analysis has been performed in both cases, only considering *X *> 0 or *X *= 0 to analyze induction and relaxation dynamics respectively.

Our method based on the analysis of nullcline's geometry allows to determine the dynamics of the system upon input as well as to determine the relaxation dynamics when the external input disappears. Initially, in absence of input the system remains stable in a fixed point determined by the crossing between nullclines in the phase space (*ϕ*_*X *= 0_). After input addition nullclines change providing a new stable fixed point (*ϕ*_*X *> 0_). The system evolves from the initial point to this new final state following a trajectory in the phase space constrained to the specific geometry of the nullclines upon input. If the external input is removed the nullclines recover the initial geometry, and hence the system evolves towards the initial stable fixed point. Here, the dynamics is constrained to the specific geometry of the nullclines under no input. However, in both cases the same mathematical analysis can be applied. For illustrative proposes a particular example is considered in figure (9). Figure (9a) shows the evolution from the initial state without input, determined by the crossing between nullclines (blue nullclines), to the new stable state upon input (brown nullcllines). The dynamics is determined by the location of the separatrix (green line) calculated upon input. In this example, the dynamics is a pulser (*P*^+ ^*T *^+^). If the input is removed, the system will return to the initial state. Figure (9b) shows the location of the separatix (grey line) calculated for the system without input, where the relaxation dynamics (green dashed line) is a grader (*G*^-^). The conditions determining the dynamics are different with (*ξ *> *ϕ*_*X *= 0 _> *S*) and without input (*ϕ*_*X *> 0 _> *S *> *ξ*) providing different dynamics. The same mathematical analysis has been applied in both cases, only considering *X *> 0 or *X *= 0 to analyze induction and relaxation dynamics respectively.

### Analysis of the dynamics in nullclines with two crossings

**Figure 10:**
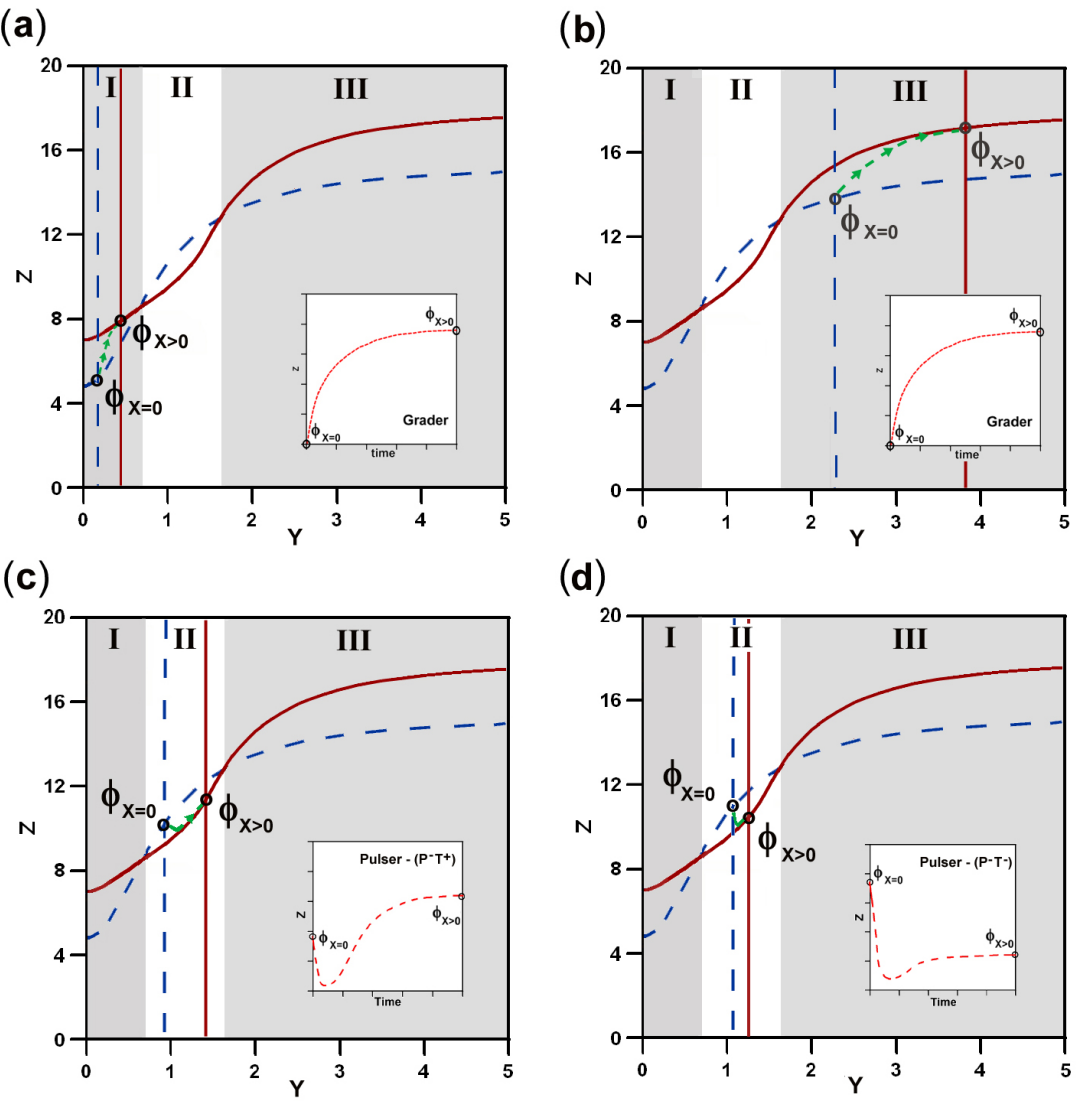
**Possible dynamics of C1 in a geometrical scenario with two crossing between nullclines**. The phase space can be divided in three different regions I, II and III delimited by the crossings between nullclines. Figures (a) and (b) have the same qualitative relations between parameters, i.e.. *ϕ*_*X *= 0 _<*ϕ*_*X *> 0_, and hence a single dynamics *G*^+ ^can emerge. Figures (c) and (d) show the two possible scenarios *ϕ*_*X *= 0 _<*ϕ*_*X *> 0 _and *ϕ*_*X *= 0 _> *ϕ*_*X *> 0_, providing *P*^-^(*T*^+^) and *P*^-^(*T*^-^) dynamics respectively. Note that in (a) and (b) the location of *ϕ*_*X *= 0 _determines the dynamics independently of the location of *ϕ*_*X *> 0_. However, scenario (c) constraints the locations of *ϕ*_*X *> 0 _to the region II. Finally, scenario (d) can be found with *ϕ*_*X *> 0 _located in region II or III. The insets represent the corresponding time courses.

In order to simplify or study, geometrical scenarios with two crossings between nullclines have been not considered due to their low probability of emergence (see main text). However, the mathematical analysis applied can be easily extended to these scenarios with double crossings. For illustrative proposes the possible dynamics of the circuit C1 with two crossing between its nullclines is analyzed. Figure (10) shows the four possible combinations of nullclines with and without input. With the same geometrical conditions determined by the value of the parameters that determine the biological interactions of the circuit, different dynamics can emerge depending on the relative location of the initial and final points. The phase space can be split into three different regions, namely I, II and III. Regions I and III show the same relative position between initial and final point, i.e. *ϕ*_*X *= 0 _<*ϕ*_*X *> 0 _(see figures (10a) and (10b)). In these scenarios, according with table 1 only *G*^+ ^dynamics can exist (independently of the location of the separatrix). On the other hand, in the region II there are two different possibilities, namely *ϕ*_*x *= 0 _<*ϕ*_*x *> 0 _(figure (10c)) or *ϕ*_*x *= 0 _> *ϕ*_*x *> 0 _(figure (10d)). In the first case, according with table 1 only *P*^-^(*T*^+^) dynamics can emerge, whereas in the second case, the unique possible dynamics is *P*^-^(*T*^-^). In summary, the example analyzed with double crossing geometry shows how the phase space can be divided in three independent regions and the possible dynamics in each region can be determined using the same analysis performed in phase space with a single crossing between nullclines.

### Backbone sequences and the associated dynamics

Within the backbone sequence, all geometrical requirements for the full description of the nullcline scenario and the resulting dynamics are listed. In the presented tables (3) and (4) all possible sequences for C4 and I1 are shown.

**Table 3:** Backbone sequences for C4

*β*^*XY*^, 1	*β *^*XY*^, *β *^*Y*^	*Z*_0_|_*X *= 0_, *Z*_*HA*_|_*X *> 0_	*Z*_0_|_*X *> 0_, *Z*_*HA*_|_*X *= 0_	Slope	*ϕ*_*X *= 0_, *ϕ*_*X *> 0_	*ϕ*_*X *= 0_, *ξ*	Relation	Function
>	*	*	*	+	*	*	*ξ *> *S *> *ϕ*_*X *> 0_	*G*^+^

>	*	<	*	-	*	*	*ξ *> *S *> *ϕ*_*X *> 0_	*G*^+^

>	*	<	*	-	*	*	*ξ *> *ϕ*_*X *> 0 _> *S*	*G*^+^

>	*	<	*	-	*	*	*S *> *ξ *> *ϕ*_*X *> 0_	*G*^+^

∅	>	>	*	*	*	*	*ξ *> *S *> *ϕ*_*X *> 0_	*G*^+^

∅	>	>	*	*	*	*	*ξ *> *ϕ*_*X *> 0 _> *S*	*G*^+^

∅	>	>	*	*	*	*	*S *> *ξ *> *ϕ*_*X *> 0_	*G*^+^

*	<	*	*	*	*	<	*ξ *> *S *> *ϕ*_*X *> 0_	*G*^+^

*	<	*	*	*	*	<	*ξ *> *ϕ*_*X *> 0 _> *S*	*G*^+^

*	<	*	*	*	*	<	*S *> *ξ *> *ϕ*_*X *> 0_	*G*^+^

*	<	*	*	*	>	*	*ϕ*_*X *> 0 _> *S *> *ξ*	*G*^-^

>	*	*	*	+	*	*	*ξ *> *ϕ*_*X *> 0 _> *S*	*P*^+^*T*^+^

>	*	*	*	+	*	*	*S *> *ξ *> *ϕ*_*X *> 0_	*P*^+^*T*^+^

*	<	*	*	*	>	*	*S *> *ϕ*_*X *> 0 _> *ξ*	*P*^-^*T*^-^

*	<	*	*	*	>	*	*ϕ*_*X *> 0 _> *ξ *> *S*	*P*^-^*T*^-^

*	<	*	*	*	<	>	*ϕ*_*X *> 0 _> *S *> *ξ*	Impossible

*	<	*	*	∅	<	>	*ϕ*_*X *> 0 _> *ξ *> S	*P*^-^*T*^+^

*	<	*	*	∅	<	>	*S *> *ϕ*_*X *> 0 _> *ξ*	*P*^-^*T*^+^

All possible backbone sequences for C4 and its relation to the circuit's function.

**Table 4:** Backbone sequences for I1

*β*^*XY*^, 1	*β*^*XY*^, *β*^*Y*^	*Z*_0_|_*X *= 0_, *Z*_*HA*_*|*_*X *> 0_	Z_0_|_*X *> 0_, *Z*_*HA*_*|*_*X *= 0_	Slope	*ϕ*_*X *= 0_, *ϕ*_*X *> 0_	*ϕ*_*X *= 0_,*ξ*	Relation	Function
>	*	*	*	+	*	*	*ξ *> *S *> *ϕ*_*X *> 0_	*G*^+^

>	*	*	*	+	*	*	*ξ *> *ϕ*_*X *> 0 _> *S*	*G*^+^

>	*	*	*	+	*	*	*S *> *ξ *> *ϕ*_*X *> 0_	*G*^+^

>	*	<	*	-	*	*	*ξ *> *S *> *ϕ*_*X *> 0_	*G*^+^

∅	>	>	*	*	<	*	*ξ *> *S *> *ϕ*_*X *> 0_	*G*^+^

*	<	*	*	*	<	*	*ξ *> *S *> *ϕ*_*X *> 0_	*G*^+^

*	<	*	*	*	*	>	*S *> *ϕ*_*X *> 0 _> *ξ*	*G*^-^

*	<	*	*	*	*	>	*ϕ*_*X *> 0 _> *ξ *> *S*	*G*^-^

*	<	*	*	*	*	>	*ϕ*_*X *> 0 _> *S *> *ξ*	*G*^-^

>	*	<	*	-	*	*	*ξ *> *ϕ*_*X *> 0 _> *S*	*P*^+^*T*^+^

>	*	<	*	-	*	*	*S *> *ξ *> *ϕ*_*X *> 0_	*P*^+^*T*^+^

∅	>	>	*	*	<	*	*ξ *> *ϕ*_*X *> 0 _> *S*	*P*^+^*T*^+^

∅	>	>	*	*	<	*	*S *> *ξ *> *ϕ*_*X *> 0_	*P*^+^*T*^+^

*	<	*	*	*	<	*	*ξ *> *ϕ*_*X *> 0 _> *S*	*P*^+^*T*^+^

*	<	*	*	*	<	*	*S *> *ξ *> *ϕ*_*X *> 0_	*P*^+^*T*^+^

∅	>	>	*	*	>	*	*ξ *> *S *> *ϕ*_*X *> 0_	Impossible

∅	>	>	*	*	>	*	*ξ *> *ϕ*_*X *> 0 _> *S*	*P*^+^*T*^-^

∅	>	>	*	*	>	*	*S *> *ξ *> *ϕ*_*X *> 0_	*P*^+^*T*^-^

*	<	*	*	*	>	<	*ξ *> *S *> *ϕ*_*X *> 0_	Impossible

*	<	*	*	*	>	<	*ξ *> *ϕ*_*X *> 0 _> *S*	*P*^+^*T*^-^

*	<	*	*	*	>	<	*S *> *ξ *> *ϕ*_*X *> 0_	*P*^+^*T*^-^

All possible backbone sequences for I1 and its relation to the circuit's function.

### Kurtosis as a measure of FFL specialization

Our results indicated that, despite the topology of FFL does not determine univocally its dynamics, it is responsible for the probability of generating a given dynamics. Some topologies exhibit higher degrees of specialization, i.e. certain dynamics are more likely than others. On the other hand, there are topologies where the probabilities of emergence of the different dynamics are more similar. In order to quantify the degree of specialization we propose to measure the kurtosis of the probability distributions for each topology. Theoretically, the maximum degree of specialization would correspond to a motif able to implement only a single dynamic, i.e. its distribution of probabilities would be single peaked, and hence display maximum kurtosis. The maximum flexibility would correspond to a system where all possible dynamics have the same probability, i.e. a flat distribution of probabilities and hence minimal kurtosis. Kurtosis is defined as the fourth standardized moment [43],

(16)

where *μ*_4 _if the fourth moment about the mean, and *σ *is the standard deviation. Here *K*_0 _is a reference value known as excess kurtosis. In general *K*_0 _= 3 in order to make the kurtosis of the normal distribution equal to zero. This allows obtaining positive kurtosis, i.e. distributions with higher *peakdeness *than the normal distribution, or negative kurtosis flatter that the normal distribution. Without lost of generality we can consider *K*_0 _= 0. In this context, kurtosis is defined positive and the kurtosis values can be directly compared: systems with higher kurtosis will have higher degree of specialization

## Authors' contributions

JM: study conception, research design, mathematical analysis, manuscript writing. SW: study conception, research design, mathematical analysis, manuscript writing. RS:study conception, manuscript writing.
